# Deletion of *QDR* genes in a bioethanol-producing yeast strain reduces propagation of contaminating lactic acid bacteria

**DOI:** 10.1038/s41598-023-32062-0

**Published:** 2023-03-27

**Authors:** George C. Kapetanakis, Luis Santos Sousa, Charlotte Felten, Loïc Mues, Philippe Gabant, Laurence Van Nedervelde, Isabelle Georis, Bruno André

**Affiliations:** 1 Molecular Physiology of the Cell, Université Libre de Bruxelles (ULB), Biopark, Gosselies, Belgium; 2 Syngulon, Seraing, Belgium; 3 BRALim (Brewing and Food Sciences), LABIRIS, Brussels, Belgium; 4 Department of Biochemical Industry, YEaST, LABIRIS, Brussels, Belgium

**Keywords:** Industrial microbiology, Fungal physiology

## Abstract

Bacterial contaminations in yeast fermentation tanks are a recurring problem for the bioethanol production industry. Lactic acid bacteria (LAB), particularly of the genus *Lactobacillus*, are the most common contaminants. Their proliferation can reduce fermentation efficiency or even impose premature shutdown for cleaning. We have previously reported that laboratory yeast strains naturally excrete amino acids via transporters of the Drug: H^+^ Antiporter-1 (DHA1) family. This excretion allows yeast to cross-feed LAB, which are most often unable to grow without an external amino acid supply. Whether industrial yeast strains used in bioethanol production likewise promote LAB proliferation through cross-feeding has not been investigated. In this study, we first show that the yeast strain *Ethanol Red* used in ethanol production supports growth of *Lactobacillus fermentum* in an amino-acid-free synthetic medium. This effect was markedly reduced upon homozygous deletion of the *QDR3* gene encoding a DHA1-family amino acid exporter. We further show that cultivation of *Ethanol Red* in a nonsterile sugarcane-molasses-based medium is associated with an increase in lactic acid due to LAB growth. When *Ethanol Red* lacked the *QDR1*, *QDR2*, and *QDR3* genes, this lactic acid production was not observed and ethanol production was not significantly reduced. Our results indicate that *Ethanol Red* cultivated in synthetic or molasses medium sustains LAB proliferation in a manner that depends on its ability to excrete amino acids via Qdr transporters. They further suggest that using mutant industrial yeast derivatives lacking DHA1-family amino acid exporters may be a way to reduce the risk of bacterial contaminations during fermentation.

## Introduction

Bioethanol is the biofuel most used worldwide in the transportation sector, its production having increased regularly since the early nineties^1^. In 2020, the USA and Brazil accounted, respectively, for 53% and 30% of global biofuel production^2^. Bioethanol is produced from a wide variety of renewable resources (feedstocks), and the yeast *Saccharomyces cerevisiae* is by far the microorganism most frequently used to carry out the biological process exploited in industrial-scale ethanol production, thanks to its unmatched ethanol yield (> 90%), productivity (> 1 g.L^−1^.h^−1^), and tolerance (> 40 g.L^−1^) and its ability to ferment a wide range of sugars^1^. In Brazil, bioethanol is produced from sugarcane molasses by high-cell-density fed-batch fermentation, in volumes reaching half a million liters. The elevated cell densities reached in this process (10% wet weight per volume) allow 6–12-h fermentations followed by recycling of the yeast biomass throughout the production season^3^. As this process is operated under non-sterile conditions, bacterial contamination occurs at high frequency^3^. Contaminants negatively impact yeast fermentation through competition for scarce nutrients and release of growth inhibitors. This generates considerable economic losses, mostly due to a reduced ethanol production efficiency and also, if the contamination is uncontrolled, to fermenter shutdown for cleaning^4,5^.

In nature, yeasts and lactic acid bacteria (LAB) are often encountered together, and metabolite exchanges between them have been reported^6,7^. Sometimes this cohabitation is desirable, as during production of fermented products such as kefir^8^ or kimchi^9^. Yet LAB are also the most common and troublesome bacterial contaminants found in ethanol production facilities, because they grow rapidly and tolerate high temperatures and low pH^10^. Besides competing with yeast for essential nutrients, LAB produce many compounds that inhibit yeast growth, including lactic acid, acetic acid, caproic acid, carbon dioxide, diacetyl, hydrogen peroxide, reuterin, phenyllactic acid, 3-carboxylic fatty acids, and cyclic peptides^11^. Historically, several methods have been applied to prevent their undesirable growth in yeast fermentation processes. In general, bacterial infections are easily controlled with antibiotics, acid treatments, ammonia, and urea-hydrogen peroxide^4,12^. Antimicrobial compounds such as c-hydroxycinnamates, organic acids, and membrane-active antimicrobial peptides^13^ have also been used, with varying degrees of success. These methods, however, pose a potential biological and environmental hazard if waste is not properly disposed of. Furthermore, some of these treatments are quite costly^14^. Hence, novel strategies are needed to decrease LAB proliferation in industrial bioethanol production plants.

LAB are typically auxotrophic for several amino acids and thus depend, for growth, on an external amino acid supply. Importantly, yeast cells grown in defined media have been found to excrete amino acids which can be used by LAB for growth^6,7^. Hence, an important and still open question is whether such cross-feeding contributes to propagation of contaminating LAB in bioethanol production systems. In yeast, excretion of amino acids is mediated by proteins of Drug:H^+^ Antiporter family 1 (DHA1), such as Aqr1, Qdr2, and Qdr3^15,16^. Importantly, we have recently demonstrated that deleting these three genes in a laboratory strain reduces the yeast’s ability to cross-feed *Lactobacillus fermentum*^16^, a LAB commonly found among contaminating bacteria in bioethanol production facilities. This observation suggests that cultivation of a bioethanol-producing yeast strain lacking specific DHA1-family transporters might be a way to reduce LAB contamination.

In this study we show that in a sterile minimal medium, the industrial yeast strain *Ethanol Red,* selected for ethanol production, sustains growth of co-cultivated *Lactobacillus fermentum* much more efficiently than a mutant derivative lacking the *QDR3* gene. We next show that growth of *Ethanol Red* on a nonsterile sugarcane-molasses-based medium is accompanied by an increase in lactic acid, and that this increase is no longer observed when the strain lacks the *QDR1*, *QDR2,* and *QDR3* genes. These results suggest that using industrial yeast strains with mutations in *QDR* genes may be a good way to reduce LAB contaminations in bioethanol-production fermentations tanks.

## Results

### The bioethanol-producing yeast strain *Ethanol Red* cross-feeds *Lactobacillus fermentum*

**Figure 1:**
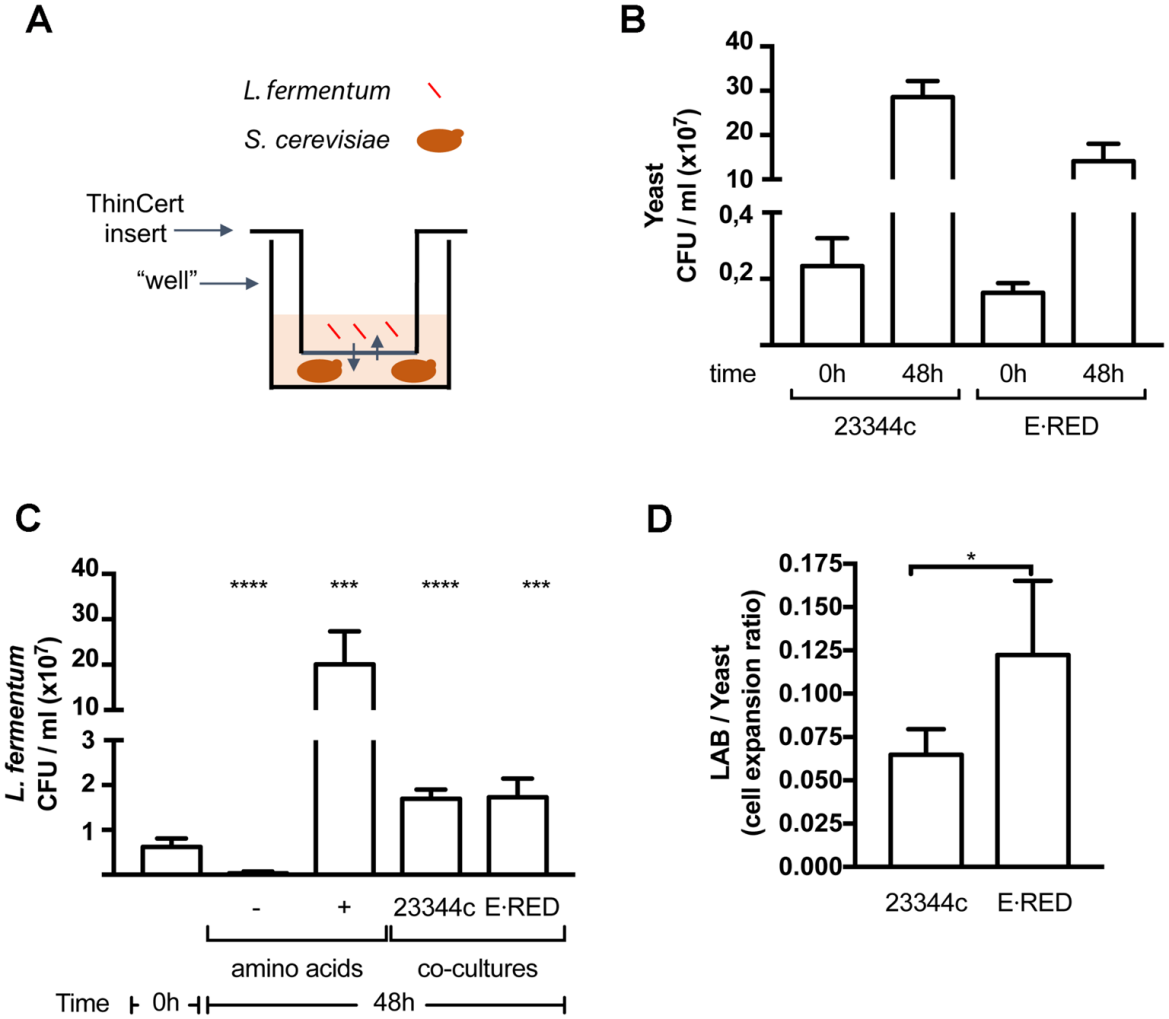
The bioethanol-producing yeast strain *Ethanol Red* cross-feeds *Lactobacillus fermentum*. (**A**) Schematic representation of the co-culture system. The yeast cell suspension is placed in the well and the insert prefilled with the *L. fermentum* cell suspension is placed on top of it. The arrows represent solute diffusion across the membrane separating the two cultures. (**B**) The density of yeast cells in cultures of the wild-type laboratory strain 23344c or the industrial strain *Ethanol Red* (E⋅RED) were monitored by counting colony-forming units (CFUs) just after inoculation (0 h) and after 48 h of co-culture. (**C**) *L. fermentum* cell density was monitored by counting CFUs in co-cultures with the 23344c wild-type or the *Ethanol Red* (E⋅RED) yeast strain, just after inoculation (0 h) and after 48 h of co-culture. In a control experiment, *L. fermentum* cell density was assessed after cultivation, in the absence of yeast, in the presence or absence of all 20 amino acids. (**D**) The values presented in (**B** and **C**) were used to calculate the cell propagation ratio of *L. fermentum* co-cultivated with 23344c or E⋅RED. Bars represent averages of minimum three independent experiments ± standard deviation (SD). * indicates a statistically significant difference as determined with the unpaired *t* test. * *P* < 0.034; *** *P* < 0.0002; **** *P* < 0.0001.

Laboratory strains of *S. cerevisiae* have been reported to naturally excrete amino acids which can be used by co-cultivated lactic acid bacteria (LAB) unable to grow without an external amino acid supply^6,7,16^. This cross-feeding can be visualized in experiments where the two microorganisms are co-cultivated in an appropriate amino-acid-free medium. For instance, we have previously reported that *L. fermentum* can grow in a MES-buffered glucose minimal medium (code number 169) containing NH_4_^+^ as sole nitrogen source, and thus in the absence of any external amino acid supply, when it is co-cultivated with a laboratory yeast strain^16^. We sought to determine whether an industrial yeast strain selected for high-efficiency ethanol production could also cross-feed *L. fermentum*. We chose the strain *Ethanol Red*, a standard in industrial biofuel production. This industrial strain was compared with our haploid wild-type reference strain 23344c (derived from the Σ1278b wild-type laboratory strain^17^), whose unique *ura3* auxotrophy was complemented by a plasmid-borne *URA3* gene. We first compared the growth of the *Ethanol Red* and 23344c strains on the MES-buffered NH_4_^+^ medium containing glucose or ethanol as carbon source. While the two strains displayed similar growth on glucose, *Ethanol Red* grew much more slowly on ethanol (Fig. S1A), in keeping with a previous study^18^. We then cultivated the cells in wells filled with buffered glucose NH_4_^+^ medium and subdivided into two compartments separated by a solute-permeable membrane (Fig. 1A). One compartment was inoculated with the 23344c or *Ethanol Red* strain and the other with *L. fermentum*. Just after inoculation and after two days of growth, culture samples were withdrawn and yeast and *L. fermentum* cell densities were quantified by counting the number of colony-forming units (CFU/ml) obtained after plating culture samples on appropriate solid rich media. After two days of cultivation, 23344c and *Ethanol Red* were found to have proliferated similarly (Fig. 1B)*. L. fermentum* had also proliferated when co-cultivated with either 23344c or *Ethanol Red* strains (Fig. 1C, D). That this growth of *L. fermentum* was due to cross-feeding by yeast cells excreting amino acids was confirmed in control experiments: the bacterium failed to grow when placed alone in the MES-buffered glucose NH_4_^+^ medium, but proliferated well if the medium was supplemented with a mix of the twenty proteinaceous amino acids (Fig. 1C). This result indicates that the *Ethanol Red* industrial yeast strain can excrete amino acids and cross-feed *L. fermentum*.

To determine which amino acid auxotrophies of *L. fermentum* are efficiently compensated by co-culture with yeast, we used the MES-buffered glucose NH_4_^+^ medium containing all twenty amino acids as a positive control and tested how omitting each amino acid individually affected *L. fermentum* growth. The bacterium was found to grow normally in the absence of alanine, asparagine, proline, glutamate, or serine (Fig. S1B), but it failed to grow in the absence of threonine, phenylalanine, methionine, glutamine, histidine, arginine, cysteine, tyrosine, leucine, lysine, tryptophan, isoleucine, or valine. In the absence of glycine or aspartate, its growth was reduced but detectable. These results indicate that both *Ethanol Red* and 23344c excrete sufficient amounts of each of the thirteen above-listed essential amino acids to support growth of *L. fermentum*.

### In the *Ethanol Red* strain, the Qdr3 amino-acid exporter contributes importantly to cross-feeding of *Lactobacillus fermentum*

**Figure 2:**
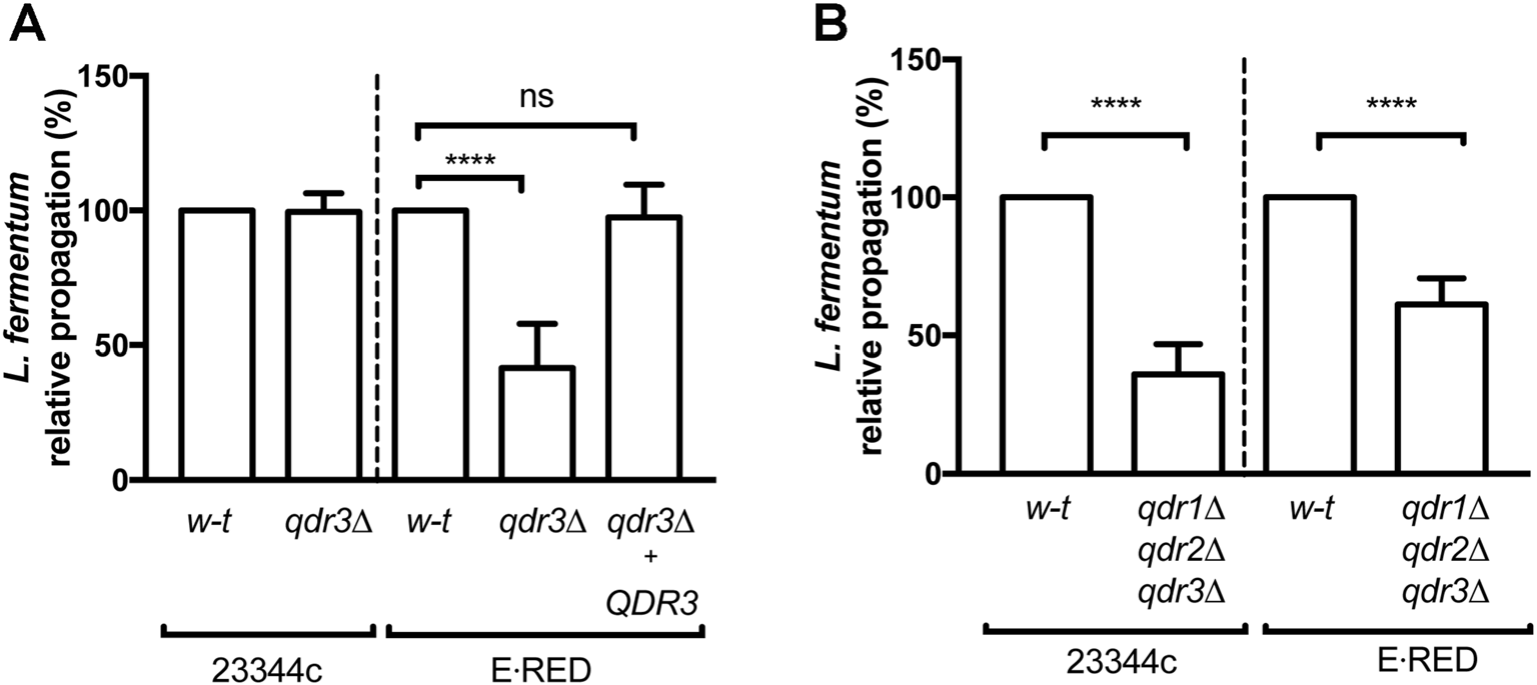
Role of Qdr amino-acid exporters in cross-feeding of *Lactobacillus fermentum*. (**A**) The values presented in Figure S2 were used to calculate the relative propagation of *L. fermentum* co-cultivated with either *Ethanol Red* (E⋅RED), its *qdr3Δ* mutant derivative, or the same mutant transformed with a plasmid carrying the *QDR3* gene from strain 23344c (right). The same experiment was performed to compare strain 23344c (*w–t*) with its *qdr3Δ* derivative mutant (left). (**B**) Same as in A, except that the values in Figure S3 were used to compare *L. fermentum* propagation when co-cultivated with *Ethanol Red* (E⋅RED) or its *qdr1Δ qdr2Δ qdr3Δ* mutant derivative (right). The same experiment was performed to compare strain 23344c (*w–t*) with its *qdr1Δ qdr2Δ qdr3Δ* derivative mutant (left). Bars represent averages of minimum three independent experiments ± standard deviation (SD). * indicates a statistically significant difference as determined with the unpaired *t* test. **** *P* < 0.0001; ns: not significant, *P* > 0.05.

In a recent study using strain 23344c as a reference wild type, we found the DHA1-family membrane transporters Aqr1, Qdr2, and Qdr3 to contribute to amino-acid excretion and cross-feeding of *L. fermentum* during growth in the MES-buffered glucose NH_4_^+^ medium^16^. To determine if this holds true for the *Ethanol Red* strain, we used CRISPR-Cas9^19^ and adapted transformation protocols to first produce a mutant derivative lacking the *QDR3* gene. We obtained many clones with heterozygous deletion of *QDR3* and a single homozygous *qdr3Δ* mutant. When we co-cultivated this mutant with *L. fermentum* in the MES-buffered glucose NH_4_^+^ medium without any added amino acid, the *qdr3Δ* strain was found to support growth of *L. fermentum*, though less efficiently than the original *Ethanol Red* strain (Fig. 2A and Fig. S2). To ascertain that this phenotype was due to the *qdr3Δ* mutation, we transformed the mutant with a plasmid bearing the *QDR3* gene, using as a selection marker a resistance gene for the antibiotic geneticin. The transformed strain was initially cultured in the presence of geneticin and then cells were collected, washed, and used to inoculate antibiotic-free minimal medium. After 48 h of co-culture with *L. fermentum* and CFU counting, the plasmid-transformed *qdr3Δ* mutant was found to support growth of the bacterium as efficiently as the *Ethanol Red* strain (Fig. 2A). Furthermore, whether transformed or not with the *QDR3*-bearing plasmid, the *qdr3Δ* mutant proliferated as well as *Ethanol Red* (Fig. S2A). In a parallel experiment, the *qdr3Δ* mutant isolated from strain 23344c likewise proliferated as well as its parental wild-type (Fig. S2B), but in keeping with previous observations^16^, the two strains showed a similar ability to support growth of *L. fermentum* (Fig. 2A). These observations thus show, unexpectedly, that Qdr3 contributes more importantly in *Ethanol Red* than in 23344c to excretion of one or several amino acids essential to growth of *L. fermentum.* It thus seems that in 23344c, other amino acid exporters can efficiently perform this excretion.

The results presented in Fig. 2A also show that the *qdr3Δ* mutant isolated from *Ethanol Red* strain can still support growth of *L. fermentum*. This suggests that excretion of amino acids required for *L. fermentum* proliferation is reduced but not abolished when Qdr3 is not functional. We thus examined whether deletion of additional amino acid exporter genes might further reduce the ability of *Ethanol Red* to cross-feed *L. fermentum*. Specifically, we applied CRISPR-Cas9 to the *qdr3Δ* mutant to delete, in a single step, the highly similar and adjacent *QDR1* and *QDR2* genes. These likely originate from a duplication event, and chromosomal synteny analysis^20^ suggests that the ancestral *QDR1/2* gene is a paralog of the *AQR1* gene encoding another DHA1-family amino acid exporter^15,16^. The derived *qdr1Δ qdr2Δ qdr3Δ* mutant strain co-cultivated with *L. fermentum* sustained growth of the bacterium as efficiently as the single *qdr3Δ* mutant (Fig. 2B and Fig. S3). In contrast, the same *QDR1* and *QDR2* deletions in the *qdr3Δ* mutant of strain 23344c resulted in decreased propagation of co-cultivated *L. fermentum* (Fig. 2B and Fig. S3). In conclusion, under the conditions used here, the Qdr3 amino acid exporter of *Ethanol Red* plays an important role in cross-feeding *L. fermentum*. Residual cross-feeding does occur, however, even if Qdr1 and Qdr2 are also lost. In laboratory strain 23344c, Qdr3 does not seem so important in supporting growth of *L. fermentum*, but additional loss of Qdr1 and Qdr2 considerably reduces cross-feeding. It thus seems that the relative contributions of the Qdr proteins to amino-acid excretion differ significantly between *Ethanol Red* and 23344c.

The *AQR1* gene encodes another well-characterized DHA1-family amino acid exporter^15,16^. It was not possible to investigate the role of Aqr1 in amino acid excretion by *Ethanol Red* because homozygous deletion of the *AQR1* gene in this strain proved much more difficult than for the *QDR* genes.

### Loss of Qdr amino acid exporters in the *Ethanol Red* strain prevents an increase in lactic acid during growth on molasses

**Figure 3:**
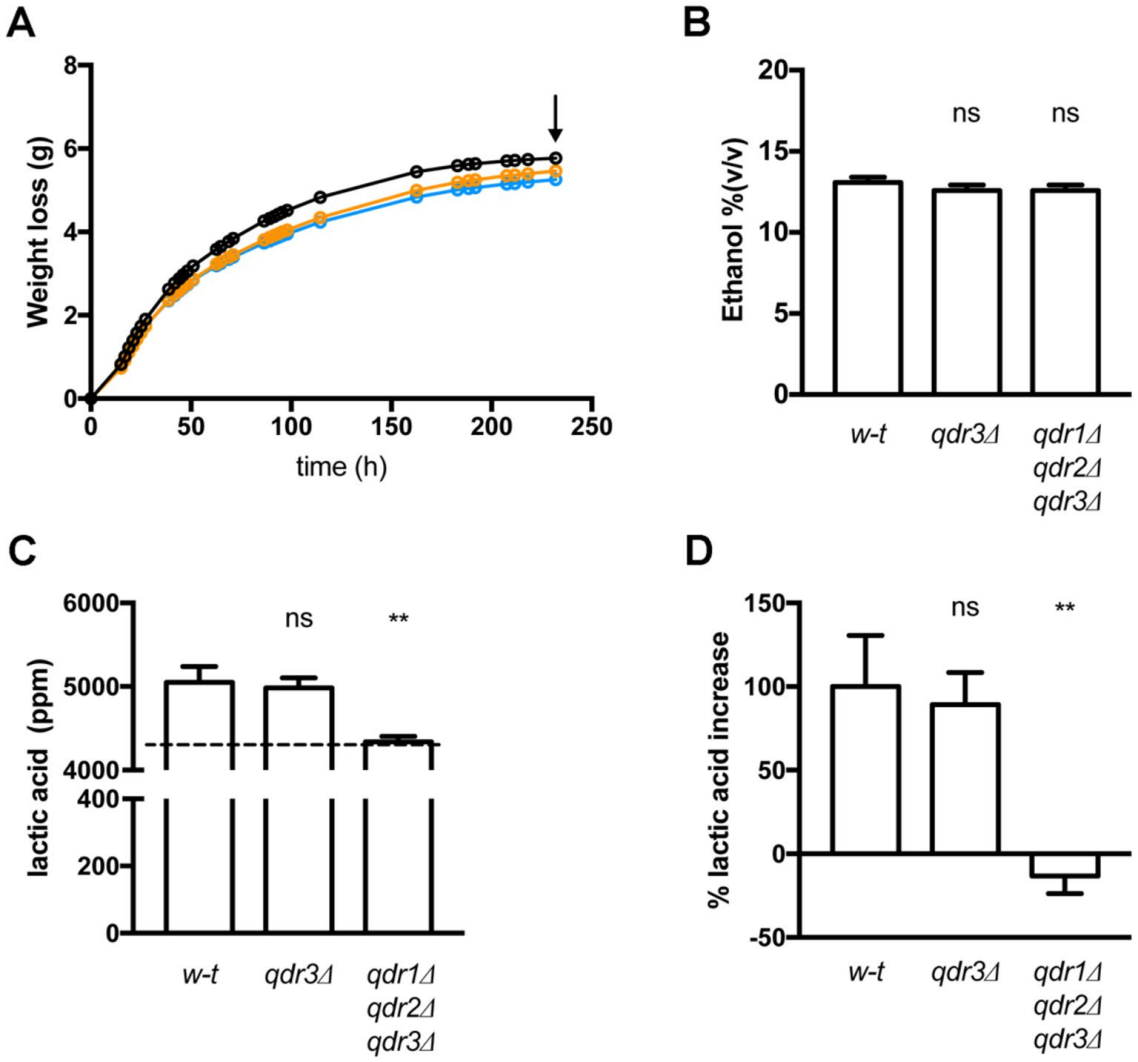
Loss of Qdr amino acid exporters in the *Ethanol Red* strain prevents a lactic acid increase during growth on molasses. (**A**) Wild-type (black)*, qdr3Δ* (orange), and *qdr1Δ qdr2Δ qdr3Δ* (blue) *Ethanol Red* cells were grown on nonsterile molasses-based medium and weight loss was monitored along time. Arrow show the end of each fermentation and the sampling timepoint. (**B**) Ethanol concentration in the medium at the end of each fermentation. (**C**) Lactic acid concentration at the end of each fermentation. The dotted line corresponds to the lactic acid detected in the medium before inoculation with yeast. (**D**) Concentrations of lactic acid expressed as percentages of that observed in supernatants of the wild-type cultures. Bars represent averages of three independent experiments ± standard deviation (SD). * indicates a statistically significant difference as determined with the unpaired *t* test. ** *P* < 0.0021; ns: not significant, *P* > 0.05.

Nonsterile crop-derived culture media used for industrial production of bioethanol generally contain several LAB species whose propagation during fermentation causes a detectable increase in lactic acid^21^. To assess the importance of amino-acid excretion by industrial yeast strains in the proliferation of these LAB, flasks containing equal volumes of a nonsterile molasses-based medium were inoculated with equivalent cell samples of the *Ethanol Red* strain or its *qdr3Δ* or *qdr1Δ qdr2Δ qdr3Δ* mutant derivatives. The culture flasks were incubated for 240 h at 30 °C with shaking and their gradual weight loss due to CO_2_ production was measured. This allowed us to compare the efficiency of glucose fermentation by the three strains, which proved reproducibly similar (Fig. 3A). Accordingly, we observed no significant difference in ethanol concentration, as measured after 240 h in several independent experiments (Fig. 3B). We also measured the lactic acid concentration in the media, before and after yeast cultivation. In cultures of *Ethanol Red*, we detected an increase in lactic acid, indicating that LAB proliferated to some extent (Fig. 3C, D). A similar increase in lactic acid was observed in cultures of the *qdr3Δ* mutant, but none was detected in cultures of the *qdr1Δ qdr2Δ qdr3Δ* mutant (Fig. 3C, D). This shows that lactic acid production was dependent on yeast and its ability to produce functional Qdr proteins. This observation, together with the results of cross-feeding experiments in defined MES-buffered glucose NH_4_^+^ medium, shows that *Ethanol Red* excretes amino acids via the Qdr exporters and that this excretion supports growth of LAB naturally present in molasses. It also suggests that deleting specific *QDR* genes in bioethanol-producing yeast strains may be a novel strategy for reducing the risk of contamination of bioethanol fermentation tanks with lactic acid bacteria.

## Discussion

**Figure 4:**
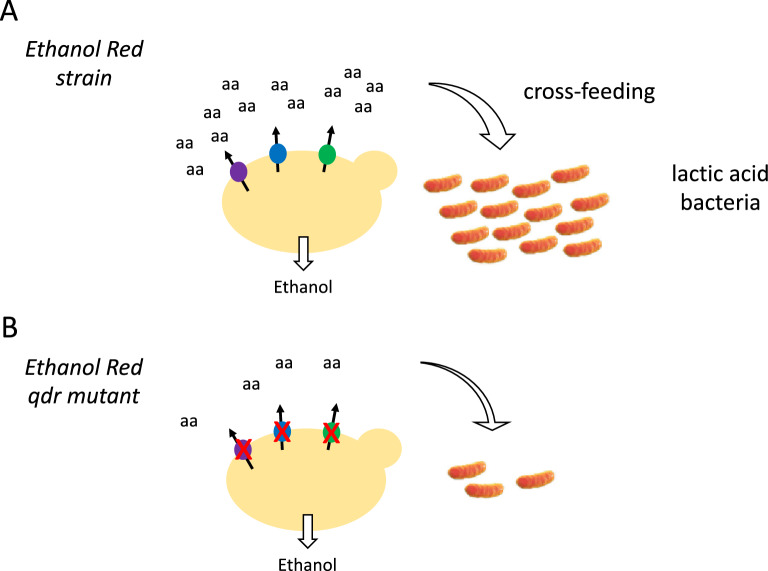
A novel strategy for reducing the risk of bacterial contaminations during bioethanol production. (**A**) The *Ethanol strain* used in this study excretes multiple amino acids via Qdr amino-acid exporters of the DHA1 family, thereby promoting proliferation of contaminating lactic acid bacteria. (**B**) Loss of Qdr transporters by means of homozygous mutations reduces amino acid excretion and cross-feeding of lactic acid bacteria, without altering ethanol production.

The proliferation of contaminating LAB in yeast fermentation tanks is a recurring problem causing a significant reduction of bioethanol production yields^5,22^. To prevent or at least reduce the risk of such contaminations at bioethanol production plants, it is imperative to better understand the factors promoting LAB proliferation in this context. Contaminating LAB are typically auxotrophic for multiple amino acids and thus require an external supply of amino acids in order to proliferate. On the other hand, it is well established that yeast cells naturally excrete several amino acids^7,23^. We have previously shown that the yeast DHA1-family transporters Aqr1, Qdr2, and Qdr3 contribute to amino acid excretion from cells^15,16^. Although why this excretion takes place remains unclear, it is known to be favored by conditions such as nitrogen overflow^7^ and/or a limited supply of other nutrients^23,24^. We therefore reasoned that this natural excretion of amino acids by yeast might contribute to LAB proliferation in nonsterile media used in industrial fermentations. To test this hypothesis, we introduced homozygous deletions of genes encoding DHA1-family amino-acid exporters into the genome of strain *Ethanol Red* used in bioethanol production. Our most important observation concerns lactic acid production, indicative of LAB proliferation, in nonsterile molasses-based medium containing either the *Ethanol Red* strain or its triple mutant lacking the *QDR1*, *QDR2* and *QDR3* genes: in contrast to cultures seeded with the parental strain, those containing the triple mutant showed no significant increase in lactic acid over a ten-day growth period. We conclude that LAB proliferation was supported by yeast-excreted amino acids rather than by free amino acids naturally available in the medium or released as a consequence of cell death. Our work further highlights Qdr1, -2, and -3 as three DHA1-family transporters that likely play a particularly important role in LAB-growth-sustaining excretion of amino acids by *Ethanol Red* (Fig. 4). To our knowledge, this has not been described before. Importantly, the absence of these proteins did not reduce this strain’s ethanol-producing capacity. This opens the prospect of exploiting yeast *qdr* mutants to reduce or control the risk of LAB contamination during bioethanol production.

To further assess the potential of this strategy, it will be useful to conduct further experiments with alternative industrial cultivation media and bioethanol-producing yeast strains. Additional work will also be needed to evaluate the respective contributions of different DHA1-family amino acid exporters to LAB cross-feeding under industrial fermentation conditions. For instance, although we show here that lactic acid was not produced by LAB in sugarcane molasses-based medium seeded with the *qdr1Δ qdr2Δ qdr3*Δ triple-mutant strain, we cannot rule out the possibility that a *qdr1Δ* or *qdr2Δ* single mutant or a *qdr1Δ qdr2Δ* double mutant might have displayed a similar advantage. This seems unlikely, however, as previous cross-feeding experiments have shown that several DHA1-family genes need to be inactivated in order to markedly reduce excretion of a single amino acid, e.g. threonine or homoserine^15,16^. Furthermore, in peculiar culture media, deletion of additional *DHA1*-family genes might be necessary to reduce amino acid excretion. In our laboratory strain, for instance, the Aqr1 transporter plays an important role in amino acid excretion^15,16^. It will thus be of interest to delete the corresponding gene in *Ethanol Red* and in its *qdr1Δ qdr2Δ qdr3Δ* derivative to see if this deletion eliminates the residual cross-feeding of *L. fermentum* observed with the *qdr1Δ qdr2Δ qdr3Δ* mutant in synthetic buffered minimal medium. For reasons that remain to be clarified, however, deleting this gene proved particularly difficult and could not be achieved in the framework of this work.

Our study further shows that in a synthetic medium, deletion of *QDR3* alone is sufficient to reduce cross-feeding of *L. fermentum* by the *Ethanol Red* and that further deletion of *QDR1* and *QDR2* does not enhance this effect. This contrasts with the results obtained with the same strains grown on the nonsterile molasses-based medium, which likely contains several LAB species: in this case, deletion of *QDR3* alone did not significantly reduce lactic acid production. Why these differences exist remains unknown. Perhaps growth on molasses medium alters the expression profile of *DHA1*-family genes in *Ethanol Red*, or perhaps the LAB species present in molasses have auxotrophies that differ from those present in *L. fermentum*. This illustrates the possibility that reducing LAB growth might require introducing different combinations of *qdr* mutations according to the culture medium used.

Reducing LAB propagation could favor the proliferation of other bacteria, for instance acetic acid bacteria^25^. In support of this view, we observed a significant increase in acetic acid production in nonsterile molasses cultures inoculated with the *qdr1Δ qdr2Δ qdr3Δ* triple mutant, as compared with the parental *Ethanol Red* strain (Fig. S4). Thus, reduced amino acid excretion by yeast might promote growth of acetic acid bacteria by causing lesser proliferation of several LAB species. Interestingly, acetate production was also observed in molasses medium seeded with the *qdr3Δ* single mutant, although lactic acid production was not reduced (Fig. S4). Perhaps the lack of Qdr3-dependent excretion of specific amino acids prevented proliferation of some but not all LAB species, this being sufficient to allow proliferation of acetic acid bacteria in the available ecological niche. This illustrates the complexity of interactions between microorganisms in co-cultures. Further investigation of these interactions and of the molecular mechanisms underlying them, including cross-feeding, should make it possible to better control bacterial proliferation during bioethanol production.

In conclusion, we report that deletion of *QDR* genes in the *Ethanol Red* strain reduces its ability to support propagation of lactic acid bacteria and provides a potentially efficient means of limiting contamination of fermentations by LAB during industrial bioethanol production. This approach can in principle be applied also to second-generation bioethanol production processes relying on nonsterile lignocellulose biomass and to industrial production by yeast of other compounds of interest.

## Methods

### Strains and growth conditions

The *Saccharomyces cerevisiae* strains used in this study (Table 1) derive from the laboratory strain Σ1278b^17^ or from the industrial strain *Ethanol Red* (Leaf, Lesaffre, Marcq-en-Baroeul, France). Cells of Σ1278b-derived strains were transformed with plasmids (Table 2) as previously described^26^. The same protocol was used to transform cells of the *Ethanol Red* strain, except that PEG4000 was replaced with PEG3350 and higher amounts of DNA (10–25 μg) were used. To introduce homozygous gene deletions in the *Ethanol Red* strain, we applied CRISPR-Cas9 as previously described^18^. Yeast and *Lactobacillus fermentum* (strain LMG17551 from the Belgian Coordinated Collections of Microorganisms) were co-cultivated on a MES-buffered, amino acid free minimal medium (code number 169) containing glucose (3%) as a carbon source and ammonium as a nitrogen source added as (NH_4_)_2_SO_4_ (0.5%), as previously described^15^. For cultures on nonsterile molasses, the growth medium consisted of sugarcane molasses (30%), (NH_4_)_2_SO_4_ (567 mg/L), and a nutrient mix (350 mg/L) (Gusmer Enterprises, LN W148320).

**Table 1:** Strains used in this study.

Strains deriving from *Σ1278b*		Origin
23344c	MATα ura3	Laboratory collection
CF180	MATα ura3 qdr1-2::loxP-HphMX-loxP qdr3::loxP-KanMX-loxP	This study
CF181	MATα ura3 qdr1-2::loxP-HphMX-loxP qdr3::loxP aqr1::loxP	This study
*Ethanol Red strain and derivatives*		
Ethanol Red®		Lesaffre
CF168	qdr3Δ/qdr3Δ	This study
CF171	qdr1Δ/qdr1Δ qdr2Δ/qdr2Δ qdr3Δ/qdr3Δ	This study

**Table 2:** Plasmids used in this study.

Code	Plasmid description	references
pFL038	CEN6 URA3	^27^
pMEL10	2 μm ampR KlURA3 gRNA-CAN1.Y	^19^
Cas9-NAT	CEN6 TEF1-Cas9 natMX6	^28^
pGK044	CEN6 QDR3-kanMX-URA3	This study

### Cross-feeding of *L. fermentum* by yeast cells

Assays for measuring cross-feeding between yeast and *L. fermentum* cells were carried out in 6-well plates equipped with ThinCert cell culture inserts (Greiner -657,640), as previously described^16^.

### Fermentation efficiency assay

Yeast cells were first pre-grown in a standard rich medium containing glucose (10%), bactopeptone (2%), and yeast extract (1%). Flasks filled with 60 ml nonsterile sugarcane-molasses-based medium were then inoculated (5⋅10^6^ cells/ml) and incubated at 30 °C with shaking. Weight loss of the flasks due to CO_2_ production was monitored until it became negligible.

### Analysis of organic acids and ethanol in culture media

Production of lactic acid and acetic acid was assayed by High Performance Liquid Chromatography (HPLC) with the LCM1 Waters Chromatographic chain (600 Pump, 2487 Absorbance Detector and Degasser, 717 Plus Autosampler) and a column (Aminex HPX-87H, Biorad) thermoregulated at 65 °C. Ethanol production was assayed by headspace Gas Chromatography (GC), with Perkin-Elmer 8000 series 2 GC instrumentation, and HS-40 Automatic Injector, and an Agilent CP- WAX 52 CB WCOT FS 50 mm × 0,32 mm column. The results are given in % ethanol v/v.

## Supplementary Information

Supplementary Information.

## Data Availability

All data are contained within the manuscript.

## Abbreviations

LAB: Lactic acid bacteria
DHA1: Drug: H^+^ Antiporter-1
