# Supplementary material for: Deletion of QDR genes in a bioethanol-producing yeast strain reduces propagation of contaminating lactic acid bacteria
Source: Sci Rep. 2023 Mar 27;13:4986. doi: 10.1038/s41598-023-32062-0 (PMC10043021; doi:10.1038/s41598-023-32062-0)
Supplement: Supplementary file 1 — Supplementary Information. [file 41598_2023_32062_MOESM1_ESM.pdf]

Deletion of *QDR* genes in a bioethanol-producing yeast strain reduces propagation of contaminating lactic acid bacteria

George C. Kapetanakis, Luis Santos Sousa, Charlotte Feleten, Philippe Gabant, Loïc Mues, Laurence Van Nedervelde, Isabelle Georis, Bruno André

Supplementary information

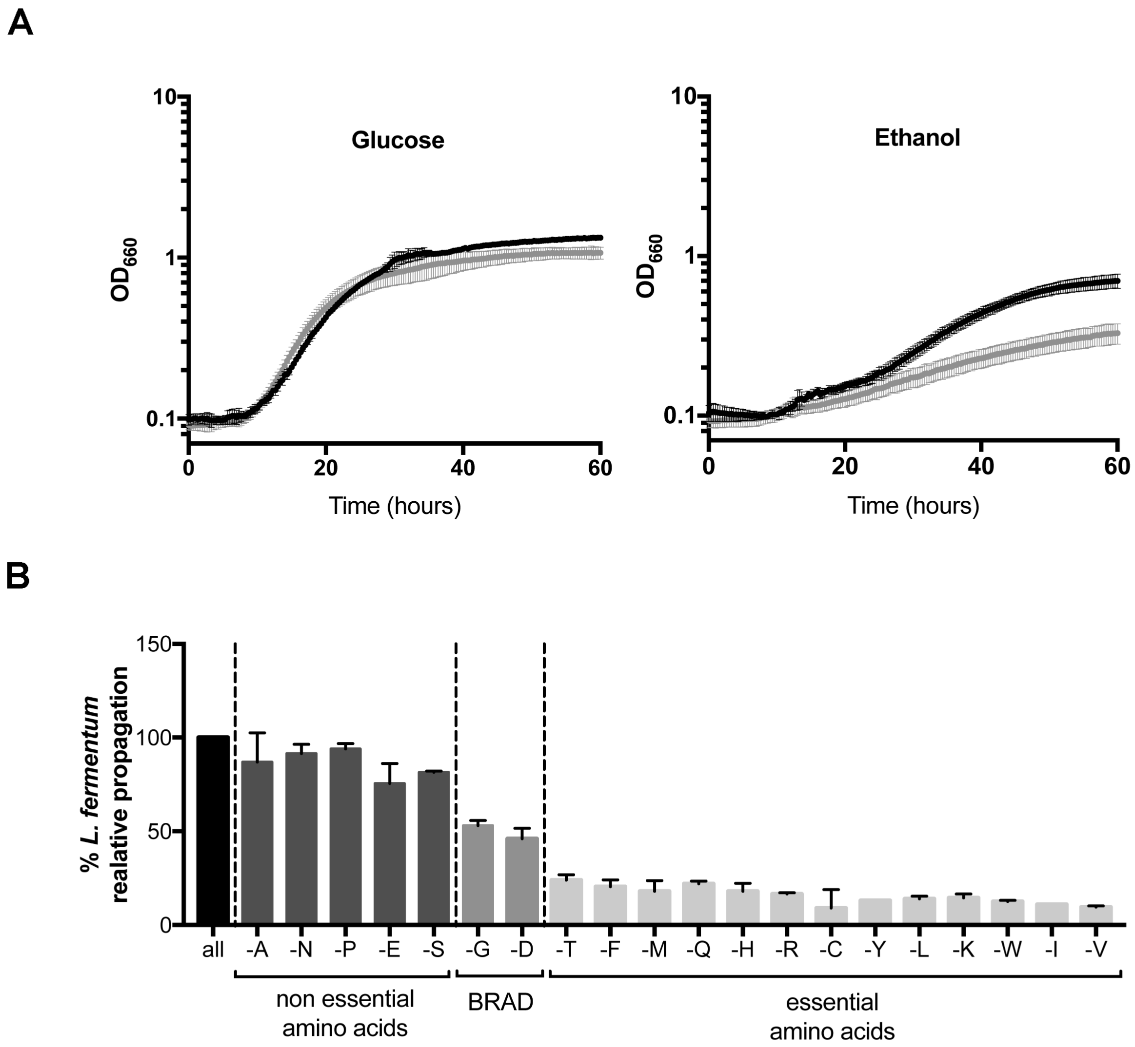

Figure S1. Growth properties of yeast industrial strain *Ethanol Red*®, yeast laboratory strain 23344c, and *Lactobacillus fermentum*. **(A)** Cells of the yeast 23344c (black) and *Ethanol Red*® (grey) strains were grown at 29°C for 60 h in multi-well plates filled with 169 medium containing ammonium (($NH4)_2SO_4$ 0,5% w/v) as nitrogen source and 3% w/v glucose or 2% v/v ethanol as carbon source. Initial optical density (OD_{660nm}) was ~ 0.05. Bars represent averages of two independent experiments ± standard deviation (SD). **(B)** *L. fermentum* is auxotrophic for several amino acids. *L fermentum* cells were initially grown in 169 ammonium glucose medium supplemented with all 20 amino acids. The cells were then washed and resuspended in the same medium (all) or an equivalent medium lacking a single amino acid (the single-letter code for each amino acid is indicated). The OD_{660nm} of the initial cultures was ~ 0.05. Cell growth was expressed as a percentage of the maximal growth observed in medium containing all 20 amino acids. Bars represent averages of 2 independent experiments ± standard deviation (SD). BRAD: amino acids for which *L. fermentum* is bradytroph.

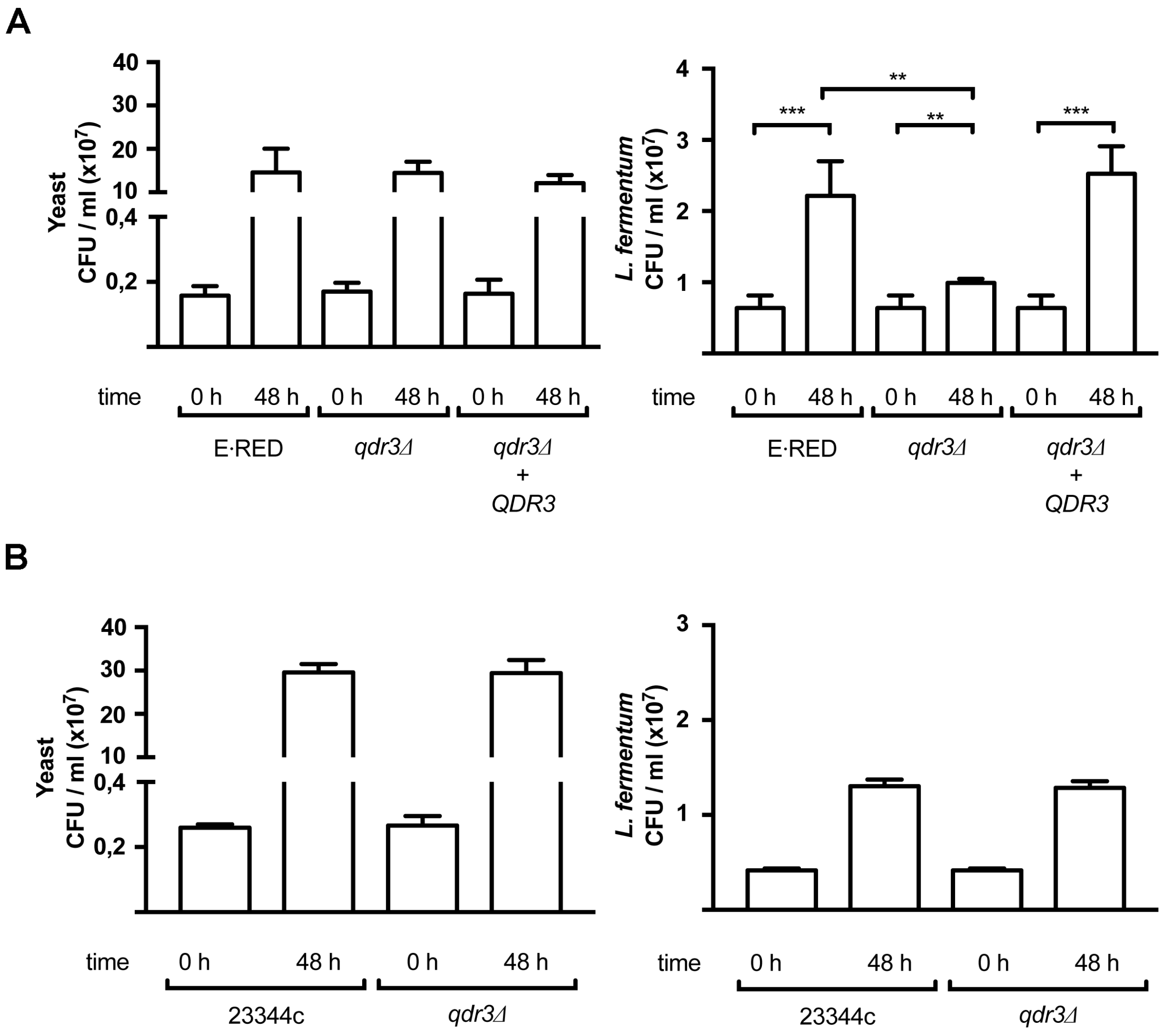

Figure S2. Cell expansion in the co-cultures presented in Figure 2A. **(A)** Cell densities of yeasts *Ethanol Red*® (E·RED), its *qdr3*Δ derivative, and the *qdr3Δ* strain harboring a *QDR3*-carrying plasmid (left panel) or co-cultivated *L. fermentum* (right panel) were assessed by counting CFUs just after inoculation (0 h) and after 48 h of co-culture growth. These values were used to calculate the relative expansion ratios presented in Figure 2A (right). **(B)** Cell densities of the laboratory wild-type yeast strain 23344c and its *qdr3*Δ derivative (left panel), or co-cultivated *L. fermentum* (right panel) were assessed by counting CFUs as in A. These values were used to calculate the relative expansion ratios presented in Figure 2A (left). Bars represent averages of minimum three independent experiments ± standard deviation (SD). * indicates a statistically significant difference as determined with the unpaired *t* test. ** P<0.0021; *** P<0.0002; ns: not significant, P>0.05.

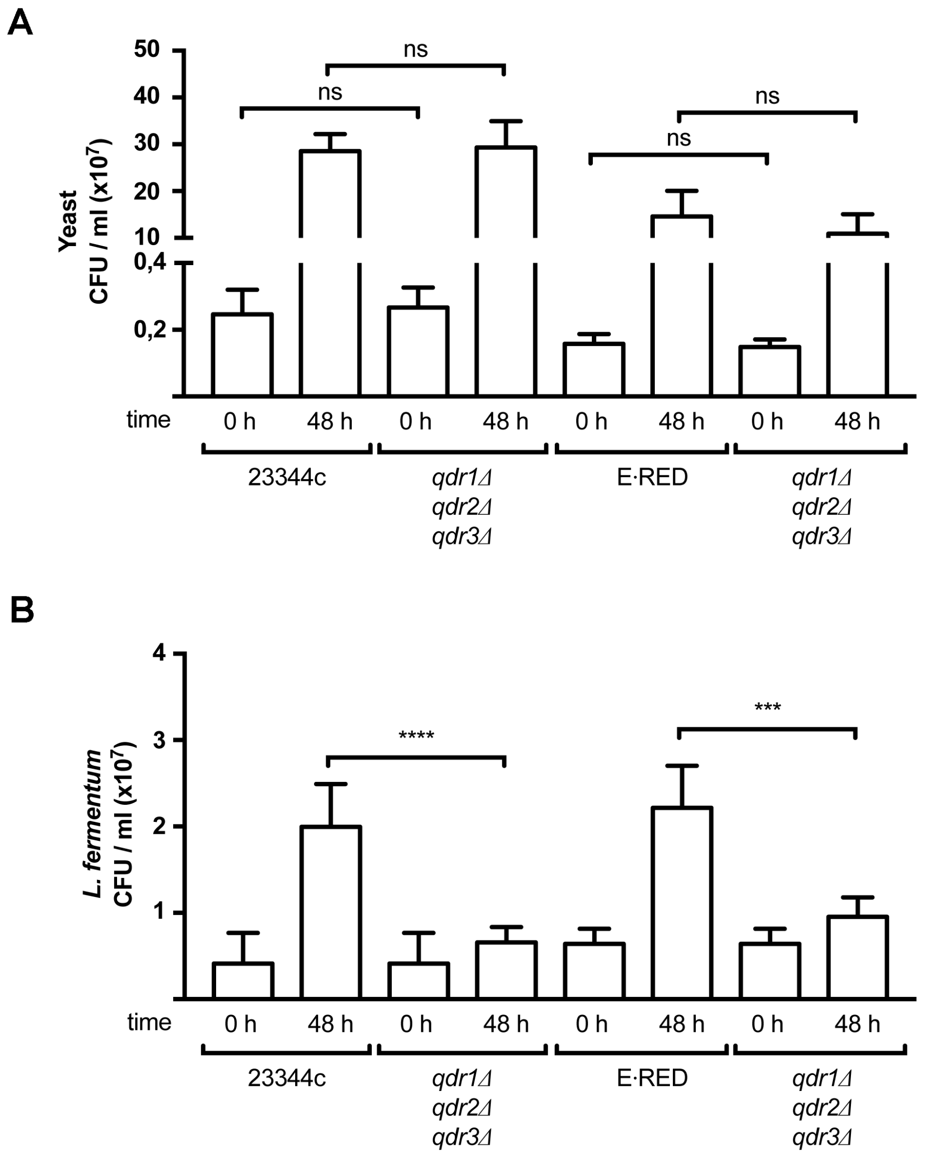

Figure S3. Cell expansion in the co-cultures presented in Figure 2B. **(A)** Cell densities of wild-type and *qdr1Δ qdr2Δ qdr3Δ* yeasts were assessed by counting CFUs just after inoculation and after 48 h of coculture. **(B)** Cell densities of L. *fermentum* cross-fed by a wild-type or *qdr1Δ qdr2Δ qdr3Δ* yeast (laboratory strain 23344c or industrial, strain *Ethanol Red*® (E·RED)) were assessed by counting CFUs just after inoculation and after 48 h of co-culture. These values were used to calculate the relative expansion ratios presented in Figure 2B. Bars represent averages of minimum three independent experiments ± standard deviation (SD). * indicates a statistically significant difference as determined with the unpaired *t* test. *** P<0.0002; **** P<0.0001; ns: not significant, P>0.05.

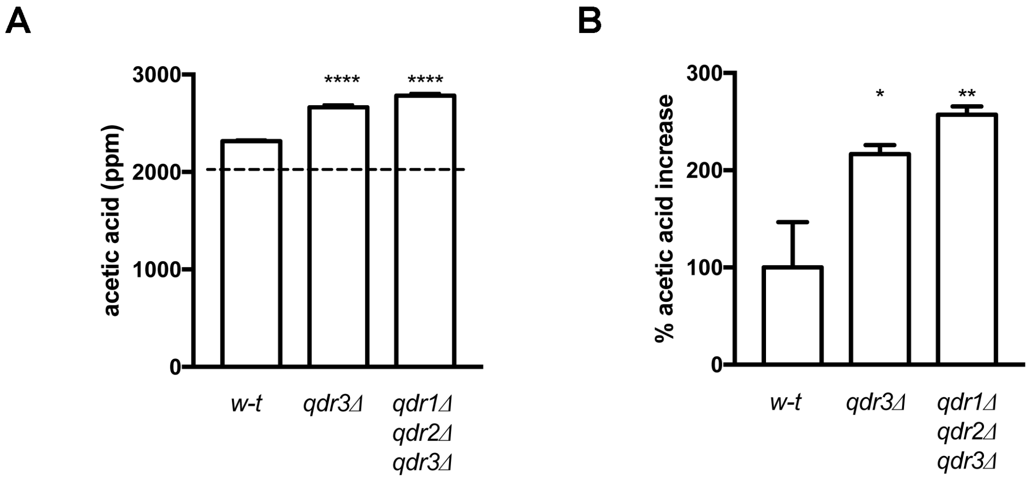

Figure S4. Acetic acid detection in cultures of *Ethanol Red*® isolates grown on molasses. (A) Wild-type*, qdr3Δ,* and *qdr1Δ qdr2Δ qdr3Δ* cells of *Ethanol Red*® were grown on molasses-based medium. Acetic acid concentration was measured at the end of the fermentation as indicated by the arrow in Figure 3A. The dotted line corresponds to the acetic acid detected before inoculation of yeast. **(B)** Acetic acid concentrations are expressed as percentages of that observed in wild-type culture supernatants. Bars represent averages of three independent experiments ± standard deviation (SD). * indicates a statistically significant difference as determined with the unpaired t-test. * $P<0.0332$; ** $P<0.0021$, ****$P<0.0001$.
